# Affective–Sexual Relationships for Money beyond Prostitution: An Analysis of the Discourse of Women Sex Workers in Chile’s Great North

**DOI:** 10.3390/ijerph182312317

**Published:** 2021-11-23

**Authors:** Jimena Silva Segovia, Pablo Zuleta Pastor, Estefany Castillo Ravanal

**Affiliations:** 1 Postgraduate and Technology Transfer Directorate, Universidad de Tarapacá, Arica 1000000, Chile; 2 Faculty of Social Sciences, School of Psychology, Universidad Bernardo O’Higgins, Santiago 8320000, Chile; pablozuletapastor@gmail.com; 3 Faculty of Humanities, School of Psychology, Universidad Católica del Norte, Antofagasta 1240000, Chile; castilloravanale@gmail.com

**Keywords:** sex workers, mining work, virility, Chile

## Abstract

A critical analysis of the discourse of female sex workers residing in the Antofagasta region in northern Chile is presented. It highlights the discursive constructions of female sex workers on the commercial, affective, and sexual bond with male mineworkers. From this discursive production emerges the image of a whore–mother, of a woman who, encouraged by a monetary transaction, knows how to embody what her client–miner needs beyond sex: to reinforce his manhood while welcoming him and recognizing him as an affectively deprived subject. Such a role fulfills a function of repairing the virile force of work, sustaining a balance that affects the miner’s functioning and performance in the mine.

## 1. Introduction

Some of the most deeply rooted conflicts in the construction of miners’ masculine subjectivities in Chile are linked to the expression of affection and emotions, an unspoken issue in the hegemonic model of masculinity, and the organization of work and production [1,2]. These conflicts impose a model based on gender imperatives and the trials of masculine subjectivity in mining work to which most workers ascribe. From this framework, these men are discursively constructed; they are situated within a context that calls for trust and homosocial alliance and shuns any hint of weakness, which is associated with feminine stereotypes.

Consequently, emotional life and the expression of emotions within this context have not been studied in depth, precisely because it is assumed that such issues are not relevant within the framework of hegemonic masculinity stereotypes [3].

Mining work is profiled internationally as an androcentric profession that mainly involves men with various socioeconomic, educational, age, and ethnic characteristics, which are organized according to hierarchies of power and command. From the gender perspective, mining work and its organization, in addition to the demanding context in which it is carried out—one that involves working in shifts at high altitudes, in extreme climates, under arid conditions, and at great distances from the family in a field with high accident rates, among other factors—exacerbates expressions of hegemonic masculinity since this model is understood as a sociocultural construction linked to the economic, political and socioaffective scenarios of identity building [4]. Thus, a gender habitus is formed [5], linked to a dominant normative order that places men not only in opposition to women but in a position of superiority. Within the concept of hegemonic masculinity, one understands “a series of discourses and social practices that seek to define the masculine term of gender within particular historical configurations, differentiating it from the experiences of men, who are not used to submitting to such a construction, and that innumerable manifest forms of resistance”. Thus, men must “become men” by assuming certain attributes and roles, which constitute social instruments to negotiate status and power. However, the process of “becoming a man” carries certain risks: violence, difficulty with expressing suffering, consumption of stimulants, excessive speeding on highways, and higher rates of suicide, homicide and femicide [6].

How women and men express their emotions concerning their sexuality and life as a couple show how each society develops its conceptions of gender based on ideas, prejudices, values, interpretations, norms, feelings, duties, and restrictions. Based on this, worldviews of gender with an ethnocentric nature are constructed [7] and create spaces where affectivity and emotions are not limited to a simple construct and play a passive role; that is, as Pineda [8] points out, “between the social and emotional worlds, there is a relationship of mutual influence”.

In the dynamics of that journey between the social and the personal, worker and man are merged into one. We speak of a subject affected and moved by these discourses at work and at the domestic level, a level that, to some extent, represents a threat to male aspirations. An example of this process is proposed by the research of Amoros et al. [9]. They report that, in the last 10 years, the presence of women in paid work has not generated visible sociocultural transformations marked by men participating equally in domestic work or in the equitable distribution of domestic work and care that involves shared responsibilities, such as caring for children, elderly people, and sick people, among others.

Regarding local views of gender, in the region of Antofagasta, certain conflicts associated with the affective–emotional and sexual lives of women and men are evident. In this scenario, the traditional solitary and homosocial life of saltpeter miners in northern Chile is repeated in the work of modern companies, with some modifications, and the system of shift work sustains the physical and emotional distance between the miners and their families, despite the purported closeness that new communication technologies can provide [10]. In mining families, affective sexual encounters, seduction, and eroticism have unique expressions marked by the exacerbation of emblems of masculinity, such as the need to have their authority recognized and reinforce a certain type of virile image. These emblems reinstate the man’s powerful position as the father–boss but at the same time make it difficult for men to express emotions in front of significant others [1,3,11,12,13]. Additionally, the mining profession is a crucial context for the construction of discourses about women; in some cases, it positions women as caregivers or administrators of the resources of the miner provider, a position in which they are kept under obedient control, while in other cases, and less frequently, women occupy positions of political power or serve as working peers in different areas. A historically relevant position for women is as a source of sexual consumption and male entertainment, meant to provide consolation for loneliness or a channel for relief and for listening to men’s feelings of being misunderstood. This situation can be illustrated in the growing number of nightclubs in the studied region consisting of bars known as shoperías, nightclubs, or cafes con piernas, where alcohol is sold and affective–sexual exchanges are provided for money. “Café con piernas” is a variety of coffee shops characteristic of Chile, which are served by young and attractive women in lingerie or semi-naked.

In this context emerges the whore, to whom lust, and sin are attributed, and who, at the same time, calls the morality of society into question. The whore breaks away from her environment, and the behaviors imagined and expected for and imposed on women: privacy, modesty, and sexual passivity [1,14,15]. In the discourse of Galindo and Sánchez [16], “whore is not a word. It is a limit, a fence built and socially sustained by everyone to keep safe what should be on the other side of that line: the untouchable wife of the mining worker, the mother of the children who wait for him to return from his shift”.

Regarding the mining man, Kraushaar [17] describes the imaginaries that have circulated about him throughout the ages: a man of a strong nature who, through his work, contributes to the economic growth of the city and the nation: “The exploitation of the country’s copper wealth weighs on their strong shoulders. Hard and arrogant, defying the scorching sun of the desert and the penetrating cold of the pampas night” [18]. This emblematic image contains historical data that intensely reflects the current context.

However, despite the above descriptions, and as we will see in the results of this research, we can approach an understanding of the links between sex workers and the mineros (mine workers)—this time, based on the women’s discourse—that calls the following constructions into question: the virility of the miner, the self-denial of his partner and even the lust attributed to the so-called whore. “Sex worker” is how the interviewees refer to themselves in terms of their trade.

First, it should be remarked that prostitution extends beyond those who perform such work and is viewed as a social institution; insults are directed at sexually active women and may even be related to men.

However, in the latter case, there is a caveat: Whore is used for women; son of a whore is used for men [19].

According to Villa [19], this is because “in our society, any type of social protest by women is attributed to excesses of their sexuality,” representing, therefore, a transgression of the order and morality that survives in the collective imaginaries through the state and its control representatives.

For these reasons, whore transcends its own significance to form a category that concerns the entire female gender and demonizes all those who emanate eroticism, as opposed to procreation and motherhood, and promiscuity as opposed to fidelity [20].

Although the object of investigation of this article is the affective–sexual relationships and transactions between sex workers and mining workers, we elaborate a discourse analysis that focuses only on the former group, based on a sample of women who perform sex work in Norte Grande, Chile. We aim to answer the following research question: What are the subjective positions in the discourse of the sex worker regarding her relationship with the mining worker, and what discourse does she develop regarding the miner’s partner?

Based on the articulation of three theoretical inputs—elements of the psychodynamics of work proposed by Dejours [21,22], the recognition of prostitution as a social institution [23] and as a global industry [24], and the problem of masculinities and the persistent threat of falling, as articulated by Gilmore [25], Badinter [26], Schneider [27] and Meler [28]—we developed a critical analysis of the discourse that allowed us to propose three main axes for reflection. The first is the speakers’ defensive strategy regarding their work: they subtract the subjectivity of tasks related to the sex trade and replace them with an action that offers not just sex but affective services. The second proposes a prostitution decentralization as the only context in which affective–sexual transactions are exchanged for money, describing the relationships between miners and their wives as another context in which this occurs. The third constructs sex work as a key function in the reproduction of the virile labor force of male miners.

## 2. Theoretical Perspective

To approach the discourse analyzed here, a theoretical trident is used. First, we turn to the psychodynamics of work and the notion of living labor developed by Dejours [22], paying particular attention to the dynamics of subjectivation at work. Second, we address the understanding of prostitution as a social institution [23] and the place of women in the tasks of (re)production of the male labor force, in which Silvia [29] calls the patriarchy of the wage. Third, we consider the understanding of masculinities as fragile identity constructions that are constantly being tested [25,28].

### 2.1. Subjectivation of Work and Defensive of the Trade

Dejours [22] defines the act of working as the act of mobilizing subjectivity within the reality of work, that is, in the face of what the task requires beyond what is prescribed and that presumes the creativity and inventiveness of the working subject. Consequently, beyond employment, work involves the subjective mobilization involved in performing a task. From this perspective, suffering is inherent in the working experience and is experienced as a demand for resolution within the reality of work.

If the proposed solution by the worker allows him or her to perform the task well, and this is recognized by his or her peers and managers, this action can be inscribed into the dimension of identity [21] and can bring pleasure and subjective development [30]. However, when this does not occur, and the suffering persists, workers must apply defensive strategies against such pain; these strategies support the worker’s participation in the work but do not manage to transform the subjective suffering, and they may even lead to a desensitization to suffering that, over time, becomes unconscious and forms a constitutive part of the psyche and body [22].

This study will investigate how the thesis proposed by Dejours is manifested in the discourse of the sex workers who participated in this research. From this perspective, the study seeks to address their mobilization of defensive strategies to sustain themselves in their work and understand how these strategies affect the speakers’ constructions and subjective positions.

### 2.2. Prostitution as a Social Institution and Women at the Service of the (Re)Production of the Male Workforce

Beyond understanding sex workers and their clients as individual subjects who engage in certain practices, it is interesting to address prostitution as a socially instituted and naturalized practice that reproduces relationships of power and domination, and today constitutes a true industry that is nonetheless still hidden because it is considered a harmful cultural practice [24].

Paraphrasing Lourau [31], we can understand this practice through the lens of social analysis. That is, we can view it as an element of social reality in which the contradictions of the system are manifested; in this case, it is a reality in which the links are not free from agendas and transactions. From this perspective, prostitution presents a paradoxical freedom in which the worker is free to start his or her own company [32], an issue that forms the basis of many understandings of prostitution as work or as an entrepreneurial exercise of agency [24]. It is important to point out that within our team, during the three years of fieldwork, have discussed our positions regarding sex work, taking into account the crisis of the homogeneity of the “woman” subject, the discourse of sex work, and the prostitutes’ movements that debate the place that feminism has built for them. We have reflected on the denomination “sex worker,” which appears as one of the ways to fight against stigmatization, and we have ascribed ourselves to the self-denomination in which they feel represented. This position has allowed us to build bridges between women working in different sectors of the sexual market (prostitutes, porn actresses, exotic dancers, and others).

In this context, the triangle of a sex worker, miner, and the miner’s wife exists; it is within this triangle that the sex worker is located and acts as an emotional hinge, and it is there that we focus our analysis. María Galindo proposes that within this space, the whore constitutes a fence that is socially constructed and sustained to safeguard what is on the other side of the fence: the untouchable wife–partner of the mining worker and the mother of his children [16]. Thus emerges the dichotomy between Eve and Mary highlighted by Rodríguez [23], in which women serve as a symbol of pleasure and sin or as a symbol of virginal holiness. Historically, this polarity of roles has been projected in northern Chile onto whores and wives, concerning mining workers [33].

From the post-Marxist feminist perspective of Silvia [29], women are the ones who, through their unpaid work, sustain and (re)produce the male labor force, returning men to the world of work with their energy replenished. In this way, unpaid, invisible work denoted as “feminine” supports the structure of capital exploitation. In this context, this study will ask about the place that the consulted sex workers build for themselves and the wives of mining workers regarding this reproductive function from the protagonists’ perspective.

In discussions about sex work, radical feminism is not linked to abolitionism, and some radical feminists, such as Despentes [34] or Rubin [35], favor of the legalization of sex work. For a certain sector of radical feminism, the oppression of women is also a repression of their sexuality, their legitimate desires, and any form of erotic expression. They believe that prostitution and pornography can be, in effect, liberating for women and that advocating for their abolition represents a new puritanical and moralistic discourse. On the other hand, liberal feminism considers the division between the public and private spheres to be appropriate. It considers that liberalism can provide a satisfactory solution to women’s problems, perhaps because reform is necessary [36]; thus, its objective is the universalization of liberal principles and values. For its part, radical feminism criticizes this division, pointing out that what sustains the social contract is a sexual contract that affirms the dominance of men over women [37]. They consider liberalism insufficient, and an obstacle to the advancement of women’s rights [38] that is intrinsically patriarchal.

### 2.3. Becoming a Man through the Denial of Emotions and the Vulnerability of the Body

Although gender, as a normatively binary anatomical distinction, is assigned to children even before birth, manhood is not guaranteed by anatomy; in contrast, one must become a man [5,25,26]. In this way, masculinity has to be produced, exposed, and demonstrated in the social scenario. In this regard, Meler [28] argues that masculinity is based on a split from femininity after childhood that represents a state of vulnerability. After this split, consciousness will ceaselessly threaten to return to femininity. Men will have to defend themselves, faced with the threat of feminization and the imminent loss of virile status.

This facet of virility is expressed through emotions, conceived as social and historical constructions of a changing nature [39]. In this framework, in contrast to the erect penis as a sword, the body is conceived as an invulnerable shield, an armor achieved through the denial and banishment of all emotions associated with the feminine [27].

However, masculinity is not obtained once and for all: it must be defended continuously because it is always on trial, always at risk of crumbling. In this regard, the world of work and its demands represents one of the tests of masculinity [40].

Consequently, it will be the object of this study to investigate the discursive constructions that sex workers produce regarding the masculinity and virility of their clients–workers–miners.

## 3. Methodological Aspects

In this research, we ask, “What are the subjective positions in the discourse of the sex worker regarding her relationship with the mining worker, and what discourse does she develop concerning the miner’s partner?” To answer these questions, we interviewed 13 women who defined themselves as sex workers. The interviews were conducted individually (three) and in two conversation groups (with five participants); the 13 women are identified below using pseudonyms. They were between 27 and 50 years old (see Table 1). All were mothers to at least one child. All were (self) recognized as independent workers, and they were of different nationalities—Chilean, Argentine, Bolivian, and Colombian.

The participants were contacted through social organizations that group sex workers and conduct STI and HIV/AIDS prevention work, with which we have collaborated since 1999, and others by snowball (Silva, 2010)—mainly those who work on the street. We coordinated three meetings of 3 h each in a miners’ union office and another for the sex workers’ organization, both places offered shelter and protection for the interview work because they were isolated from other activities. In agreement with the women, compensation for their time was a manicure service and workshops on the body, violence, and sexuality. Free conversations were established about their experiences, under an extensive guide, for example, their opinions about the miners’ clients, the ideas about wives or partners, the miners’ searches in the sex trade, and their own emotions in these relationships. Another way to safeguard the ethics of the process is through the signing of an informed consent form that describes the objectives and purpose of the research; this is given to the participants, and they can review it before signing. The entire research procedure is reviewed and approved by members of the Ethics Committee of the Chilean National Council For Research, Science, Additionally, Technology (Conicyt).

The stories presented in these interviews were analyzed by applying the theory of critical discourse analysis [41,42,43], consisting of the sustained reading of 140 pages of text with the individual and group narratives of the seven participants in this article (documentary corpus). Subsequently, discursive fragments were selected for theoretical analysis, in which a matrix facilitating the analysis was used, which seeks to understand what the speakers do with what they say, noting what they say and what is subverted concerning the dominant discourses. We also analyzed subjective positioning in the discourse [44], which identified the positions that the speakers construct for themselves and other actors or social objects.

The procedure was as follows:

1. Review of the complete documentary corpus: interviews, conversation groups, workshops.
2. Interpretation and organization of the material by categories of analysis; in this case, according to the main discursive axes.
3. Development of analytical matrices.
4. Editing following the three main discursive axes and the theoretical perspective of the study.

## 4. Analysis and Results

### 4.1. Making Myself into Someone Else So I Can Be There

In dialog with the theoretical perspective explained above, we propose that a first discursive construction is produced through the defensive strategies that sex workers develop to face the demands of their trade, a strategy that (dis)places them from their intimate being and (re)situates them as a character.

It is an act of speech through which speakers seem to extract their subjectivity from the work situation. The following quote illustrates:

Actually, for me—and I am very sincere—it is my nourishment. My body and my face and my contact lenses are my presentation to a strange person, totally strange. It is my way of being and my way of acting, my conversation…All that is Rosita. It does not go to the person who is here at this moment; that is, it is like another me. I become another person. I transform. My contact lenses are my first transformation before a client; I feel totally inhibited if I do not have these contact lenses (Tatiana, 39 years old, Peruvian).

For Rosita, the exercise of the sex trade is the food of work. This discourse shows the (re)construction of a linear relationship between work and livelihood in which work is limited to an instrumental function; a means of obtaining sustenance. What matters is the end and not so much the means, even though, in this case, it involves the body itself in a key location. The body, however, acts as another self; it is transformed into another person. This strategic resource is understood as a defense of one’s subjectivity during the work.

Rosita proposes that in her work, she is another person; according to that logic, she uses another name for herself at work. In her experience, the demands of work, of encountering and having sex with a complete stranger, force her to be there without being there, to put another person there instead: “It is like another me. I become another person. I transform myself”. For this transformation, her main resource is contact lenses. Let us consider Dejours’ statement that the essence of subjectivity “consists of the fact of experiencing oneself” [22]. We can consider that the subject changes her eyes to transform her view of herself and the world, which enables her to perform the work and act, in her own words, “uninhibited”. Without those lenses, without that artifact that transforms the person looking and what she sees, altering her subjectivity, it is less possible to engage in the exercise of sexual commerce. The crux of the work is acting as a way of protecting one’s subjectivity at work.

Rosita’s story continues below:

Right now [during the interview], I am wearing my contact lenses, but for aesthetic reasons. However, I am not in the other me; I am speaking from myself, from what this person feels when serving a client.

The discourse explicitly distinguishes the person, the one who feels, from her other self, the character who acts. The person who feels and the character who acts are divorced in this discursive construction. We understand this divorce in a Dejourian way as a defensive strategy against work demands; it seems to speak of a subjectivity that transits back and forth from the character that must be adopted to perform the task.

On the other hand, the perspective of Sandra offers a viewpoint in which the action performed is produced discursively according to a (de)sexualized logic, since its staging emphasizes affective rather than sexual components. This is evident in the following quote:

[What I offer is] being treated well. That is, good treatment, predisposition. Eyes open, listening to what they have come to say; beyond looking for sex, they often come to vent. Ninety percent of the time, they come to let off steam, tell me their problems, their experiences, perhaps the same thing they would say when coming home. From “I am tired, I had a lot of trouble” to hoping for affection, a caress, and a kiss; affectionate treatment (Sandra, 45 years old, Bolivian).

According to this proposition, Sandra is above all a predisposed woman; that is, she is positioned, above all, to satisfy the man’s need for affective–emotional discharge as well as a sexual outlet; she constructs this role discursively as the offer of an outlet and, in turn, an opportunity for respite. In this way, the link between sex workers and clients is discursively understood as a vital pause; their transaction offers a respite that clients—in this case, male miners—seem unable to find elsewhere. We can say, then, that the speakers’ discourse places them in a position of mitigating some psychological suffering of their clients and takes the focus away from the sexual dimension of the transaction.

The whore, who is often designated as an evil woman, a public woman, emerges here as capable of containing the personal discomfort of her clients and, incidentally, fulfilling a role historically attributed to the wife. Beyond sex, the body of the sex worker seems prepared/made to listen, and—according to an interviewee—the emotional containment of her clients frames this dynamic 90% of the time.

Additionally, I say, “Hello, my love; hello, my darling; hello, baby”. That is how I treat them, right? Additionally, when you treat him better on the phone, the more he wants the service. So I say, “Look, my love; I work independently. I am on such-and-such street”. They say, “What do you look like?”. Additionally, I say, “Look, I am tall, blonde; I am a foreigner”. “…Additionally, your services?…” [they ask]. In other words, they want to know how you are going to treat them. When they go to the apartment—because it is an apartment where I meet them; it is not private—they arrive, and I say to them, “Okay, oral sex, vaginal sex, caresses, kisses, boyfriend treatment…”. “What is a boyfriend treatment?” [they ask]. “Treating me like your girlfriend” (the participants laugh—in a mocking tone)… As if they are looking for more affection from us. They want you to become their girlfriend. So they are there with the boyfriend deal. So I am already being super affectionate, super accommodating, like they are already going to that fantasy. Then, I arrive and say, “Hello, my love”; I grab him, kissing him. So what do they like? That I treat them as if I were their girlfriend; that affection. Additionally, I am super affectionate. Therefore, I have many clients from the mine. They come and go and tell me… “Are they single, married, widowed, left or separated?”, I say to them (laughs) (…) Additionally, they feel very important [in] that moment there (Maritza, 37 years old, Colombian).

The discursive construction of the client–man–worker–miner that Maritza produces is that of an affectively deprived subject, or at least, one who requires time and space to feel important and well treated; she makes it clear that she knows what men need and what they want; therefore, what is sold before sex, is what she calls the boyfriend deal. Whether her clients are single, married, widowed, or abandoned, Maritza is situated in a place that assumes knowledge about them, namely, that they want affection and caring, and not necessarily sex. This affection is required from a woman who discursively produces and advertises herself as beautiful, resolute, autonomous, and independent; has her own apartment; and—this seems important—is a foreigner, and therefore, possesses qualities brought from other locations.

Therefore, it is important to understand how this boyfriend treatment has been incorporated into the offer that foreign sex workers provide and to discern whether this has been a product of the dynamics of the Chilean sex trade or has originated from the consumers’ demands. In this sense, the word “pololo (boyfriend)” is used as the axis since it is a Chileanism that refers to a romantic bond between two people who intend to establish a long-term relationship but does not imply progression to something with a larger connotation, such as a courtship or marriage.

These sexual–affective encounters are not private, local encounters; they take place in Maritza’s apartment. The offer of sexual services seems to be more complex to the extent that the woman is enlarged, while, at the same time, the client is called a baby. In this regard, although the baby signifier is used regularly by foreign women in a loving tone, it is also true that it refers to a small child who needs care. Interestingly, according to this discursive construction, the client likes to assume the position of a baby and seeks this treatment. We are encouraged to propose that this strategy—that is, putting the male in the position of a child who will be pampered with affection—serves to (de)sexualize the activity or, at least, to (de)centralize sexual intercourse as work.

We emphasize that the enunciation of the boyfriend deal is followed by laughter with a mocking tone among the participants of the conversation, which exposes that it is an illusion that is sold to men, since the deal is to act as a boyfriend, knowing that they are not. In this way, the image for sale of a fantasy that goes beyond the purely sexual is constructed. The illusion is of making others believe and feel that they are important, or at least to offer them a time when they can experience themselves as someone important, who deserves to be cared for and treated with love. As presented in Maritza’s discourse, this performance makes it possible for clients to come and go and for the sex worker to be successful because she has interpreted the client’s needs and, perhaps, a profound reason for her recourse to prostitution.

a. The Whore is the Wife: The Circularity of Love Transactions

The discursive construction that we have been producing in this analysis, which consists of the subversion of the role commonly attributed to sex workers and the (re)significance of their work providing love and understanding, is continued in the way that the self-described sex workers characterize the wives of men–workers–miners. It is imperative here to consider that this is the discourse produced by the sex workers interviewed, interpreted as a way of constructing subjective positionings both for their mineworker clients and for their wives, which will not necessarily coincide with the latter’s enunciations:

The wives think only of the ATM (they all laugh). It has to be said… two-week periods and no more (Jasmine, 30 years old, Argentina).

In this discursive construction, the women–wives are placed in a position intertwined with their economic interest in their partners, who are, in turn, signified with the image of the automatic teller machine (ATM). According to interviewees, the objectified figure is that of the man–worker–miner, who is reduced to the function of providing money. According to the statements of the women interviewed, among those who trade affection and sex for money, wives take the lead.

Additionally, the group’s laughter during the discourse is interesting, especially because it was followed by the phrase “it has to be said”. The narrative aims to reveal a kind of unspoken truth: that women–wives only have sex “every two weeks” when the husband–man–worker–miner brings money home and exercises the cashier role. In other words, the discursive construction of the sex worker proposes that sex is also traded for money in the miner’s marital life.

In the discursive constructions of the sex workers, the man objectified by the figure of the cashier is emptied of subjectivity, recognition, and affection, and that is precisely what he seeks from the sex trade, as the following quote suggests:

So what are they looking for? Affection, because at home, they say they do not get it. The women are more dedicated to shopping than to the babies, and when they are home, they do not give their men enough time. They seek affection, attention, so that is what I, for example, give as Rosita. I give him affection, that attention: “My love, do you want some water? Do you want a drink? Do you want this?” (Rosita, 39 years old, Chilean).

The discursive construction of the woman–wife seems to be precisely that of *(in)disposition*, of a woman who is not there for the man–miner–husband and the satisfaction of his sexual or affective needs. This woman is preoccupied with caring for the children, the house, or even herself, buying things, and spending the money generated by the husband. The man turned into a cashier is left in a position of affective and sexual deprivation. The sex worker occupies this empty place. She gives him affection, attention; calls him “my love”.

Consequently, the places and roles of the wife and prostitute are shaken by these discourses; they are set in motion and are endowed with subjectivity and possibilities for action. In contrast, the man–worker–miner is located in an objectified place. Male domination is reversed, and the three vertices of the triangle are (re)signified. In this scheme, the sex worker is (self)constructed discursively as the provider of care, attention, and affection, all in exchange for money, of course. It is a love that is animated by pay that is expressed in this condition. On the other hand, the woman–wife emerges in this discourse as cold and calculating, since the husband is important to her only once every two weeks and by virtue of the fact she can extract money from him then. In this sense, to satisfy the affective–sexual needs of her husband, the woman–wife is also characterized mockingly, mobilized around an objective that is also built animated by money and is not love. Finally, the man–worker–miner, who is supposedly a representative of male domination, is emptied of subjectivity. He ends up instrumentalized by the woman–wife and excited by the sex worker. In both cases, the love he seeks is the product of action and economic transactions. It is worth explaining that this is the discursive construction of the sex workers who participated in the research and not that of the men–workers–miners or their wives, who are brought to the analysis from the imaginary and via the voices of the sex workers.

Following this line, in these voices, the miner’s family is constructed discursively as a structure in which the mine worker is paradoxically undermined. This can be interpreted from the following quote:

It is he [the mining worker] who supports the family, but no one gives value to that, neither the children nor the wife. Therefore, they try to seek this attention elsewhere. Their morale is on the floor (Anayensi, 28 years old, Colombian).

We emphasize here the signifier of value, or at least it’s dual significance. First, it is understood as the value associated with economic contribution, since without the money that the man–worker–miner earns through his work, his family is not supported and falls apart. Second, it is understood as the value associated with an ethical notion and (self) valuation, as expressed in the quote through the signifier of morality.

According to the fragment of the sex worker’s narrative presented above, the lack of recognition of the value of the man’s contribution to the house is associated with a devaluation of the subject himself since his morality ends up on the floor.

The discourse of the sex workers who participated in this research is consistent with the proposal raised by Martynowskyj [45] regarding problematizing prostitution as the exclusive domain of affective–sexual transactions for money. From this perspective, the sex trade is (re)situated as a fundamentally emotional space for the psychological restitution of working men. This can be paraphrased as follows: What sex workers provide is attention, which can be understood as an act of concern, or even respect and affection, for the other. This attention is something that this other cannot find elsewhere, although it is still attention motivated by money:

[When the money exchange occurs], for us, [it is] exciting! Because, well, it is silver (money). Without money, there is no love (Vania, 36 years old, Venezuelan).

These analyses put us on the track toward the third discursive axis, which is the objective of this analysis that seeks to understand the discursive construction of the man–worker–miner in the utterances of the sex worker.

### 4.2. Wounded Masculinities and Reparative Action

The image of wounded masculinity was developed throughout the present analysis. Therefore, we will start by returning to the last fragment quoted, which alludes to the demoralization of the man–worker–miner. In this context, we consider a third meaning of the expression “morality” and propose that what gets “floored” is male virility. This virilis virtus, addressed by Bourdieu [5], is a virility that urgently needs to be rescued. To this end, the reparatory action of the women who participated in this research is a fundamental act, as we explain in the following interview fragment:

Consuelo (50 years old, Colombian): They worry whether or not the woman climaxes, that she always has an orgasm; that is, it’s like they feel macho when the woman finishes. They say, “I made her finish”, or “I was with a woman”, or “With this one, like this: Aaah! Additionally, I made her come like a man”.

Estrella (55 years old, Ecuatorian): Additionally, because we women are so resourceful, we always make them think they did.

Cinthia (40 years old, Colombian): Because otherwise, we would still be there. They never leave.

Yisela (31 years old, Bolivian): They believe everything, then. However, no…

Danna (37 years old, Argentina): I do not know if they believe it. I think they believe in the moment, because when they ejaculate, and they are there, they say to you, “Oh, my love, how beautiful; it’s all finished”. Additionally, one says: “Oh yes, my life! (participants laugh)”. It’s just that you have to tell him… Actually, for example, I have clients that I already know; that is, in general, I already know my clients. I already know which one, for example (…) They visit me often, and those I know, generally, I already know that one likes it when I have an orgasm and what they like to do. Additionally, if they are a new customer, I try to find out.

Oriana (34 years old, Venezuelan): The truth is, the only thing I want when they arrive is for them to get on and then leave. I do not want anything else (Excerpt from group interview).

The specific sexual dimension of the relationship emerges in this excerpt, which shows that much of what men–workers–miners pay for is to feel like men.

The male in this discussion proves himself by giving the female an orgasm: “Additionally, I made her come like a man” is what Rosita interprets as the internal sensation of her client, which is satisfaction, because this is what the men are looking for, and the women know it. At the same time, the word like a preceding the word man is highlighted; it implies a sort of man, but not necessarily a true man. Moreover, although we are not interested here in a discussion about true manhood, the like a is remarkable to the extent that it follows, in this discursive construction, the action of Rosita, who, knowing what the client wants and needs—namely, to affirm his virility—feigns an orgasm. Through this action, the wounded virility of the man–worker–miner is repaired, even if the action is illusory.

Another important nuance in this action is provided by the defensive strategy described by the speaker: At the same time that she satisfies the client’s need to repair his virility (with an orgasm), she also lightens her workload, and with this, gains time away from work: by pretending to finish, the man also finishes.

In this act, the man seems to be more focused on performance than on pleasure, and his behavior centers around a primary objective: To make the woman have an orgasm and, as a result, experience himself as a man capable of pleasuring a woman. However, for his counterpart, the trick is simple: “‘Oh yes, my life!’, you have to tell him…”. Rosita leads him to believe she has had an orgasm to leave him satisfied. In this sense, it seems that the woman’s sexual expertise is not the most important thing for the man; instead, it is whether she has an orgasm that marks the self-evaluation of his performance. According to the speaker, customers return for this reason: They know that with her, they will be able to uphold a virile image.

The conclusion is clear: Regardless of the action, it is marked as the truth that the only thing she wants is for the man to leave in all senses: to ejaculate and then leave the apartment.

This analysis offers a problematization of the understanding of male clients of prostitutes. In contrast to the proposal of Volnovich [46], who argues that men seek to relate to degraded sexual objects as a way of unleashing their appetites for domination, the image that emerges from the discourses of the participants in this study is of a degraded man who seeks to repair his virile image of himself. A framework of moralities is woven in the social imaginary that presents sex workers as tainted and outcast subjects. If they do not comply with this shattered image—that is, if they do not complain about the damage that has been done to them in their lives if they do not recount how they have been forced—and instead promote themselves as figures with power or autonomy, it has a high cost to the bond they want to establish with the client who comes to be repaired. Such an attitude does not produce fear that they will not survive an attack; on the contrary, it produces fear that they are saying that the work is not as scary as it seems, and they are at ease with it [34].

## 5. Conclusions

In general terms, the discursive productions of the female speakers who participated in this research seem to be oriented toward constructing protective positions of their subjectivity that endow them with a certain degree of agency. Let us consider which, in our opinion, are the most important:

First, they are not the ones who engage in the sex trade; instead, it is someone else; a character they create, a kind of alter ego. Second, they seem to position themselves not so much to be caught but to welcome a man who seeks their services. Third, for them, affective–sexual transactions for money are not the exclusive exercise of the sex trade; they also apply to the conjugal relationships of mining workers. Fourth, they do not experience themselves or their characters as degraded sexual objects but rather as agents who claim and repair the virile subjectivity of their clients.

We propose that from the analyzed discursive productions emerges the image of a whore–mother, a woman who, encouraged by a transaction for money, knows how to embody what her client–man–worker–miner needs beyond sex: she can repair his virility while welcoming him and recognizing him as an affectively deprived subject.

Finally, we will say that being a whore–mother operates, largely, as a form of reparative work for what we will call here a virile workforce. We propose that the use of the sex trade by men–workers–miners is key for allowing them to return to work with their virile image more or less replenished, feeling capable of sustaining their performance and doing their work. From this perspective, although the analyzed discourse subverts the symbolic places and positions adopted in the sex worker–miner–miner’s wife triangle, it also seems to maintain a balance that almost directly affects the operation and performance of the miner at the worksite.

We consider that putting these experiences in debate favors the visualization of the experiences of this group of sex workers, enabling their voices in dialogue with critical theoretical approaches, placing them in the debate about their place in the sociocultural life, in this case, of the miners. We seek to contribute to a displacement of absolute positions in this context, installing their experience in the critique as a fundamental tool.

Finally, it will be important to read this article in combination with our study that focuses on analyzing the discourse of the mineworkers, wondering about the subjective positions that it constructs. In this regard, we can say that three significant constructions exist that enter into dialogue and tension with the discourse analyzed here. Namely: the image of the miner as a highly productive subject, but who at the same time is brutalized in the exercise of his work; the image of a mine worker who, in order to sustain his productivity, requires a wife who takes care of everything at home, reproducing a strict sexual division of labor that has, as a counterpart, a sort of de-eroticization of the conjugal bond, and finally, the feeling of being strangers in their own families; and, the subjective positions constructed for sex workers as distractions, in the rhetorical figures of trophy women, women who flatter and women who listen. Although subjective positions produced by their discourse are different from those produced by the sex workers’ discourse, mainly because they place themselves in a place of high productivity and heroism, it can be argued that they reproduce the same underlying idea: the use of the sex trade is key to sustaining the high productivity of the male miner.

## Figures and Tables

**Table 1:** Participants in the study.

	Code	Age	Workplace	Socioeconomic Level	Nacionality	No. of Children
1	Vania	36	In night club	Low	Venezuelan	1
2	Jasmine	30	In night club	Low	Argentina	1
3	Consuelo	50	In night club	Low	Colombian	3
4	Sandra	45	Independent	Low	Bolivian	2
5	Estrella	32	In street	Low	Ecuatorian	2
6	Anayensi	28	In street	Low	Colombian	2
7	Maritza	37	In private	Low	Colombian	1
8	Rosita	39	In private	Low	Chilean	2
9	Tatiana	29	Independent	Low	Peruvian	1
10	Cinthia	40	Independent	Low	Colombian	1
11	Yisela	31	In apartament	Low	Bolivian	1
12	Danna	37	In private	Low	Argentina	1
13	Oriana	34	In private	Low	Venezuelan	1

Own elaboration.

## Data Availability

The data supporting the findings of this study are available on request from the corresponding author (J.S., FONDECYT 1180079). The data are not publicly available due to their containing information that could compromise the privacy of research participants.
